# The decision sampling framework: a methodological approach to investigate evidence use in policy and programmatic innovation

**DOI:** 10.1186/s13012-021-01084-5

**Published:** 2021-03-11

**Authors:** Thomas I. Mackie, Ana J. Schaefer, Justeen K. Hyde, Laurel K. Leslie, Emily A. Bosk, Brittany Fishman, R. Christopher Sheldrick

**Affiliations:** 1 Department of Health Behavior, Society, and Policy, Rutgers School of Public Health, 683 Hoes Lane West, Piscataway, NJ USA; 2 Institute for Health, Health Care Policy and Aging Research, 112 Paterson Ave, New Brunswick, NJ USA; 3 Rutgers School of Public Health, 683 Hoes Lane West, Piscataway, NJ USA; 4 VA Center for HealthCare Organization and Implementation Research, Boston University School of Medicine, 72 East Concord St, Boston, MA USA; 5 American Board of Pediatrics, 111 Silver Cedar Court, Chapel Hill, NC USA; 6 Tufts School of Medicine, 35 Kneeland Street, Boston, MA USA; 7 Rutgers School of Social Work, 390 George Street, New Brunswick, NJ USA; 8 Department of Health Law, Policy and Management, School of Public Health, Boston University, One Silber Way, Boston, MA USA

**Keywords:** Health care policy, Mental health care policy, Decision-making, Decision sciences, Evidence, Foster care

## Abstract

**Background:**

Calls have been made for greater application of the decision sciences to investigate and improve use of research evidence in mental health policy and practice. This article proposes a novel method, “decision sampling,” to improve the study of decision-making and research evidence use in policy and programmatic innovation. An illustrative case study applies the decision sampling framework to investigate the decisions made by mid-level administrators when developing system-wide interventions to identify and treat the trauma of children entering foster care.

**Methods:**

Decision sampling grounds qualitative inquiry in decision analysis to elicit information about the decision-making process. Our case study engaged mid-level managers in public sector agencies (*n* = 32) from 12 states, anchoring responses on a recent index decision regarding universal trauma screening for children entering foster care. Qualitative semi-structured interviews inquired on questions aligned with key components of decision analysis, systematically collecting information on the index decisions, choices considered, information synthesized, expertise accessed, and ultimately the values expressed when selecting among available alternatives.

**Results:**

Findings resulted in identification of a case-specific decision set, gaps in available evidence across the decision set, and an understanding of the values that guided decision-making. Specifically, respondents described 14 inter-related decision points summarized in five domains for adoption of universal trauma screening protocols, including (1) reach of the screening protocol, (2) content of the screening tool, (3) threshold for referral, (4) resources for screening startup and sustainment, and (5) system capacity to respond to identified needs. Respondents engaged a continuum of information that ranged from anecdote to research evidence, synthesizing multiple types of knowledge with their expertise. Policy, clinical, and delivery system experts were consulted to help address gaps in available information, prioritize specific information, and assess “fit to context.” The role of values was revealed as participants evaluated potential trade-offs and selected among policy alternatives.

**Conclusions:**

The decision sampling framework is a novel methodological approach to investigate the decision-making process and ultimately aims to inform the development of future dissemination and implementation strategies by identifying the evidence gaps and values expressed by the decision-makers, themselves.

**Supplementary Information:**

The online version contains supplementary material available at 10.1186/s13012-021-01084-5.

Contributions to the literature

- Calls have been made for greater application of the decision sciences to remedy the well-documented challenges to research evidence use in mental health policy and practice.
- Grounded in the decision sciences, we propose a novel methodological approach, referred to as the “decision sampling framework,” to investigate the decision-making process, resulting in identification of a set of multiple and inter-related decisions, evidence gaps across this decision set, and the values expressed as decision-makers select among policy alternatives.
- The decision sampling framework ultimately aims to inform the development of future dissemination and implementation strategies by identifying the evidence gaps and values expressed by the decision-makers, themselves.

## Research article

Implementation science has been defined as “the scientific study of methods to promote the systematic uptake of research findings and other evidence-based practices into routine practice, and, hence, to improve the quality and effectiveness of health services.” [1, 2]. As implementation science grows in mental health services research, increasing emphasis has been placed on promoting the use of research evidence, or “empirical findings derived from systematic research methods and analyses” [3], in system-wide interventions that aim to improve the emotional, behavioral, and mental health of large populations of children, adolescents, and their caregivers [1, 2]. Examples include the adoption of universal mental health screening programs [4], development of trauma-informed systems of care [5], and psychotropic prescription drug monitoring programs [6–8]. Emphasis on research evidence use to inform system-wide interventions is rooted in beliefs that the use of such evidence will improve effectiveness, optimize resource allocations, mitigate the role of subjective beliefs or politics, and enhance health equity [9]. At the same time, documented challenges in the use of research evidence in mental health policy and programmatic innovation represent a sustained challenge in the implementation sciences.

To address these challenges, calls have been made for translational efforts to extend beyond simply promoting research evidence *uptake* (e.g., training the workforce to identify, critically appraise, and rapidly incorporate available research into decision-making [10–12]) to instead consider the knowledge, skills, and infrastructure required to inform and embed research evidence use in decision-making [13]. In this paper, we argue to engage the decision sciences to respond to the translational challenge of research evidence use in system-wide policy and programmatic innovations. System-wide innovations are studied far less frequently than medical treatments and often rely on cross-sectional or quasi-experimental study designs that lack a randomized control [10], adding to the uncertainty of the evidence base. Also, relevant decisions typically require considerations that extend beyond effectiveness alone, such as whether human, fiscal, and organizational capacities can accommodate implementation or provide redress to health inequity [13]. Accordingly, studies of research evidence use in system-wide policy and programmatic innovation increasingly emphasize that efforts to improve research evidence use require access not only to the research evidence, but also to the expertise needed to address the uncertainty of that evidence, and consideration for how values influence the decision-making process [14, 15].

In response, this article proposes the decision sampling framework, a systematic approach to data collection and analysis that aims to make explicit the gaps in evidence available to decision-makers by elucidating the decisions confronted and the role of research evidence and other types of information and expertise. This study specifically contributes to the field of implementation science by proposing the decision sampling framework as a methodological approach to inform the development of implementation strategies that are responsive to these evidence gaps and to inform the development of future dissemination and implementation strategies.

This article first presents our conceptualization of an optimal “evidence-informed decision” as an integration of (a) the best available evidence, (b) expertise to address scientific uncertainty in the application of that evidence, and (c) the values decision-makers prioritize to assess the trade-offs of potential options [14]. Then, we propose a specific methodological approach, “decision sampling,” that engages the theoretic tenets of the decision sciences to direct studies of evidence use in system-wide innovations to an investigation of the decision-making process itself. We conclude with a case study of decision sampling as applied to policy regarding trauma-informed screening for children in foster care.

## Leveraging the decision sciences to define an “Evidence-informed Decision”

Foundational to the decision sciences is expected utility theory—a theory of rational decision-making whereby decision-makers maximize the expectation of benefit while minimizing risks, costs, and harms. While many theoretical frameworks have been developed to address its methodological limitations [16], expected utility theory nevertheless provides important insights into optimal decision-making when confronted with the real-world challenges of accessing relevant, high-quality, and timely research evidence for policy and programmatic decisions [17]. More specifically, expected utility theory demonstrates that evidence alone does not answer the questions of “what to do;” instead, it suggests that decisions draw on evidence, where available, but also incorporates expertise to appraise that evidence and articulated values and priorities weighed in consideration of the potential harms, costs, and benefits associated with any decision [14]. Although expected utility theory—unlike other frameworks in the decision sciences [18]—does not address the role of human biases, cognitive distortions and political and financial priorities that may also influence decision-making in practice, it nevertheless offers a valuable normative theoretical model to guide how decisions might be made under optimal circumstances.

### Moving from research evidence use to knowledge synthesis

Based on a systematic review of research evidence use in policy [17], Oliver and colleagues argue that “much of the research [on policymakers’ use of research evidence] is theoretically naïve, focusing on the uptake of research evidence as opposed to evidence defined more broadly” [19]. Accordingly, some have suggested that policymakers use research evidence in conjunction with other forms of knowledge [20, 21]. Recent calls for “more critically and theoretically informed studies of decision-making” highlight the important role that broader sources of knowledge and local sources of information play alongside research evidence when making local policy decisions [17]. Research evidence to inform policy may include information on prevalence, risks factors, screening parameters, and treatment efficacy, which may be limited in their applicability to large population groups. Evaluations based on prospective experimental designs are rarely appropriate or possible for policy innovations because they do not account for the complexity of implementation contexts. Even when available, findings from these types of studies are often limited in their generalizability or not responsive to the information needs of decision-makers (e.g., feasibility, costs, sustainability) [14]. Accordingly, the research evidence available to make policy decisions is rarely so definitive or robust to rule out all alternatives, limiting the utility of research evidence alone. For this reason, local sources of information, such as administrative data, consumer and provider experience and opinion, and media stories among others, are often valued to provide data that may facilitate tailoring available research evidence to accommodate differences in heterogeneity of the population, delivery system, or sociopolitical context [20, 22–27]. Optimal decisions thus can be characterized as a process of synthesizing a variety of types of knowledge beyond available research evidence alone.

### The role of expertise

Expertise is also needed to assess whether available research evidence holds “fit to context” [28]. Local policymakers may review evidence of what works in a controlled research setting, but remain uncertain as to where and with whom it will work on the ground [29]. Such concerns are not only practical but also scientific. For example, evaluations of policy innovations may produce estimates of average impact that are “unbiased” with respect to available data yet still can produce inaccurate predictions if the impact varies across contexts, or the evaluation estimate is subject to sampling errors [29].

### The role of values in assessing population-level policies and trade-offs

Values are often considered to be antithetical to research evidence because they introduce subjective feelings into an ideally neutral process. Yet, the decision sciences suggest that values are required alongside evidence and expertise to inform optimal decision-making [30]. Application of clinical trials to medical policy offers a useful example. Frequently, medical interventions are found to reduce mortality but also quality of life. Patients and their physicians often face “preference sensitive” decisions, such as whether women at high risk of cancer attributable to the BRCA-2 gene should choose to undergo a mastectomy (i.e., removal of one or both breasts) at some cost to quality of life or not to undergo the invasive surgery and increase risk for premature mortality [31]. To weigh the trade-offs between competing outcomes—in this case the trade-off between extended lifespan and decreased quality of life—analysts often rely on “quality adjusted life years,” commonly known as QALYs. Fundamentally, QALYs represent a quantitative approach to applying patients’ values and preferences to evidence regarding trade-offs in likelihood and magnitude of different outcomes.

Likewise, policies frequently are evaluated on multiple outcome criteria, such as effectiveness, return on investment, prioritization within competing commitments, and/or stakeholder satisfaction. Efforts to promote evidence use, therefore, require consideration of the values of decision-makers and relevant stakeholders. Federal funding mechanisms emphasizing engagement of stakeholders in determining research priorities and patient-centered outcomes [32–34] are an acknowledgement of the importance of values in optimal decision-making. Specifically, when multiple criteria are relevant, (e.g., effectiveness, feasibility, acceptability, cost)—and when evidence may support a positive impact for one criteria but be inconclusive or unsupportive of another—preferences and values are necessary to make an evidence-informed decision.

To address these limitations, we recommend a new methodology, the decision sampling framework to improve analysis of decision-making to capture its varied and complicated inputs that are left out of current models. Rather than relying solely on investigation of research evidence uptake alone, this approach anchors the interview on the decision itself, focusing on respondents’ perceptions of how they synthesize available evidence, use judgment to apply that evidence, and apply values to weigh competing outcomes. By specifically orienting the interview on key dimensions of the dynamic decision-making process, this method offers a process that could improve implementation of research evidence by more accurately reflecting the decision-making process and its context.

## Case study: trauma-informed care for children in foster care

In this case study, our methodological approach focused on sampling a single decision per participant regarding the identification and treatment of children in foster care who have experienced trauma [35]. In 2016, just over 425,000 U.S. children were in foster care at any one point in time [36]. Of these, approximately 90% presented with symptoms of trauma [37], or the simultaneous or sequential occurrence of threats to physical and emotional safety including psychological maltreatment, neglect, exposure to violence, and physical and sexual abuse [38]. Despite advances in the evidence available to identify and treat trauma for children in foster care [39], a national survey indicated that less than half of the forty-eight states use at least one screening or assessment tool with a rating of A or B from the California Evidence Base Clearinghouse [4].

In response, the Child and Family Services Improvement and Innovation Act of 2011(P.L. 112-34) required Title IV-B funded child welfare agencies to develop protocols “to monitor and treat the emotional trauma associated with a child’s maltreatment and removal from home” [40], and federal funding initiatives from the Administration for Children and Families, Substance Abuse and Mental Health Services Administration (SAMHSA) and the Center for Medicare and Medicaid Services incentivized the adoption of evidence-based, trauma-specific treatments for children in foster care [41]. These federal actions offered the opportunity to investigate the process by which states used available research evidence to inform policy decisions as they sought to implement trauma-informed protocols for children in foster care.

In this case study, we sampled decisions of mid-level managers seeking to develop protocols to identify and treat trauma among children and adolescents entering foster care. Protocols specifically included a screening tool or process that aimed to inform determination of whether referral for additional assessment or service was needed. Systematic reviews and clearinghouses were available to identify the strength of evidence available for potential trauma screening tools [42, 43]. This process explicitly investigated the multiple inputs and complex process used to make decisions.

## Methods

Table 1 provides a summary of each step of the decision sampling method and a brief description of the approach taken for the illustrative case study. This study was reviewed and approved from the Institutional Review Board at Rutgers, the State University of New Jersey.

**Table 1:** Steps for the decision sampling framework and an illustrative case study

Methodological approach	Decision sampling framework	Illustrative case study
1. Identify the policy domain and scope of inquiry	a. Articulate the system-wide innovation or policy of explicit interest	Decisions sampled included related to the development, implementation, or sustainment of universal screening programs that sought to identify and treat the trauma of children entering foster care
	b. Identify the scope of the decisions relevant to the policy or programmatic domain of interest	The scope included decisions about system-wide interventions that sought to identify and treat trauma of children and adolescents entering foster care.
2. Select appropriate qualitative method and develop data collection instrument	a. Identify research paradigm and qualitative method appropriate for the research question and decision-making process	The illustrative case study engaged a post-positivist orientation to the research question posed. Due to the exploratory nature of this project, 60-min semi-structured interviews were conducted
	b. Develop interview guide that explicitly crosswalks with core and relevant constructs from the decision sciences, anchoring the interview on the discrete decision point	Cross-walking with tenants of decision analysis, developed a 26 question interview guide that included questions in following domains: • Decision points • Choices considered • Evidence and expertise regarding chances and outcomes • Outcomes prioritized (which implicitly reflect values) • Group process (including focus, formality, frequency, and function associated with the decision-making process) • Explicit values as described by the respondent • Trade-offs considered in the final decision
3. Identify sampling framework	a. Identify key informants who participate in decision-making processes for selected system-wide innovation or policy	Decisions were sampled from narratives provided by mid-level administrators from Medicaid, child welfare, and mental health agencies with roles developing policy for the provision of trauma-informed services for children in foster care. Our team then employed a key informant framework for sampling, asking to recruit individuals into the study who participated in recent decisions regarding efforts to build a trauma-informed system of care for children in foster care
	b. Sample one or more decisions from each informant	Informants were asked to describe a recent and important decision relevant to implementation. Our approach sampled a single decision per participant
4. Conduct semi-structured interview	a. Develop and execute study-specific recruitment	Recruited study participants: The team initially contacted a public sector mid-level manager in the child welfare agency who was an expert in developing protocols for provision and oversight of mental health services for children in foster care [44]. This initial contact assisted the research team in acquiring permission for participation through the respective child welfare agency
	b. Execute study-specific data collection	Semi-structured interviews were conducted over the phone by at least two trained members of the trained research team trained, including (1) a sociologist and health services researcher (TM) and (2) public health researchers (BF, AS, ER); interviews were approximately 60 min in length and were recorded with notes taken by one researcher and memos written by both researchers immediately following the interview. Of those recruited into this study, one individual declined participation. Primary data collection for the semi-structured interviews and member-checking group interviews occurred between January 2017 and August 2019
5. Conduct data analysis	a. Engage a modified framework analysis (see the “Methods” section, analytic approach)	Employed modified framework analysis, engaging the seven steps (as articulated in the Methods, analytic approach) by trained investigators (TM, AS, ER, BF), including: • Trained analyst checked transcripts for accuracy and de-identified • Trained analysts familiarized themselves with data, listening to recordings and reading transcripts • Reviewed interview transcripts generating codebook and conducted interdisciplinary team reviews employing emergent and a priori codes, with coding consensus. Entered codes into a mixed methods software program, DeDoose.^TM^ • Developed matrix to index codes thematically • Summarize excerpted codes for each thematic area • Provide excerpted data to support thematic summary in the decision-specific matrices • Systematically analyze across matrices by decision point
6. Conduct data validation	a. Engage strategies to validate analyses and reduce investigator-introduced biases (e.g., member-checking focus groups)	Conducted member-checking group interviews with a subset of decision-makers who participated in phase 1 semi-structured interviews • Identified and recruited sample: participants were selected among the 32 decision-makers who spoke to screening and assessment in their phase 1 interviews. Respondents were selected because they brought lived experience that would facilitate assessing the utility of the model given prior experiences in relevant decision-making. A total of 8 decision-makers participated in 4 group interviews • Developed and conducted group interview guides. • Group interview participants were provided a standardized slide set that: • Introduced the purpose of the study and summarized the qualitative findings from the decision sampling framework • Presented a Monte Carlo simulation model and results that were developed to synthesize available information analytically and facilitate conversation • After presentation of the decision sampling findings, respondents were asked “Does this match your experience?”, “Do you want to change anything?”, and “Do you want to add anything?” • Conducted analyses: each member-checking group interview transcript was analyzed following completion [45, 46]. We used an immersion-crystallization approach in which two study team members (TM, AS) listened to and read each group interview to identify important concepts and engaged open coding and memos to identify themes and disconfirming evidence [45, 46]. The study team members used open-codes to gain new insights about the synthesized findings from the group interviews. After the initial analysis and coding was complete, the researchers re-engaged the data to investigate for disconfirming evidence. Throughout this process, the researchers sought connections to identify themes through persistent engagement with the group interview text and in regular discussion with the interdisciplinary team

### Sample

We posit that decision sampling requires the articulation of an innovation or policy of explicit interest. Drawing from a method known as “experience sampling” that engages systematic self-report to create an archival file of daily activities [45], “decision sampling” first identifies a sample of individuals working in a policy or programmatic domain, and then samples one or more relevant decisions for each individual. While experience sampling is typically used to acquire reports of emotion, “decision sampling” is intended to acquire a systematic self-report of the process for decision-making. Rationale for the number of key informants should meet relevant qualitative standards (e.g., theoretic saturation [46]).

In this case study, key informants were mid-level administrators working on public sector policy relevant to trauma-informed screening (rather than random selection [47]); we sampled specific decisions to gain systematic insight into their decision-making processes, complex social organization, and use of research evidence, expertise, and values.

### Interview guide

**Fig. 1:**
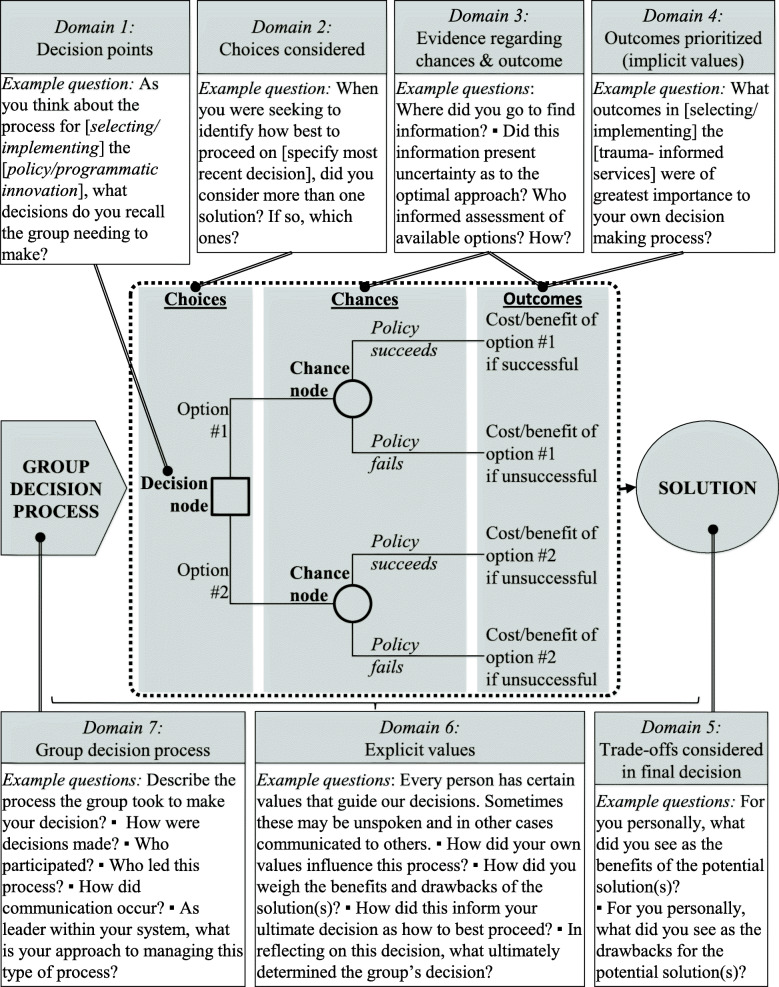
Interview Guide Domains: Mapping Interview Questions onto a Decision Analysis Framework

Rather than asking directly about evidence use, the decision sampling approach seeks to minimize response and desirability bias by anchoring the conversation on an important decision recently made. The approach thus (a) shifts the typical unit of analysis in qualitative work from the participant to the decision confronted by the participant(s) working on a policy decision and (b) leverages decision sciences to investigate that decision. Consistent with expected utility theory and as depicted in Fig. 1, the framework for an interview guide aligns with the inputs of a decision tree reflecting the choices, changes, and outcomes that are ideally considered when making a choice among two or more options. This framework then generates domains for theoretically informed inputs of the decision-making process and consideration of subsequent trade-offs.

Accordingly, our semi-structured interview guide asked decision-makers to recall a recent and important decision regarding a system-wide intervention to identify and/or treat trauma of children in foster care. Questions and probes were then anchored to this decision and mapped onto the conceptual model. As articulated in Fig. 1, domains in our guide included (a) decision points, (b) choices considered, (c) evidence and expertise regarding chances and outcomes [20, 48], (d) outcomes prioritized (which implicitly reflect values), (e) the group process (including focus, formality, frequency, and function associated with the decision-making process), (f) explicit values as described by the respondent, and (g) trade-offs considered in the final decision [48]. By inquiring about explicit values, our guide probed beyond values as a means to weigh competing outcomes (as more narrowly considered in decision analysis) to include a broader definition of values that could apply to any aspect of decision-making. Relevant sections of the interview guide are available in the supplemental materials.

### Data analysis

Data from semi-structured qualitative interviews was analyzed using a modified framework analysis, involving seven steps [49]. First, recordings were transcribed verbatim, checked for accuracy, and de-identified. Second, analysts familiarized themselves with the data, listening to recordings and reading transcripts. Third, an interdisciplinary team reviewed transcripts employing emergent and *a priori* codes—in this case aligned with the decision sampling framework (see a–g above [20, 50]). The codebook was then tested for applicability and interrater reliability. Two or more trained investigators performed line-by-line coding of each transcript using the developed codebook. The codes were then reconciled against each other arriving at consensus on the codes line-by-line. Fourth, analysts chose to use a software, a program to facilitate data indexing and a matrix, developed to index codes thematically, by interview transcript, to facilitate a summary of established codes for each decision. (The matrix used to index decisions in the present study is available in the supplement.) In steps five and six, the traditional approach to framework analysis was modified by summarizing the excerpted codes for each transcript into the decision-specific matrices with relevant quotes included. Our modification specifically aggregated data for each discrete decision point into a matrix providing the opportunity for routine and systematic analysis of each decision according to core domains. In step seven, the data were systematically analyzed across each decision matrix. Finally, we performed data validation given potential for biases introduced by the research team. As described in Table 1, our illustrative case study engaged member-checking group interviews (see supplement for the group interview guide.)

## Results

Table 2 presents informant characteristics for the full sample (in the 3rd column) as well as the subsample (in the 2nd column) who reported decisions relevant to screening and assessment that are analyzed in detail below. The findings presented below demonstrate key products of the decision sampling framework, including the (a) set of inter-related decisions (i.e., the “decision set”) routinely (although not universally) confronted in developing protocols to screen and assess for trauma, (b) diverse array of information and knowledge accessed, (c) gaps in that information, (d) role of expertise and judgment in assessing “fit to context”, and ultimately (e) values to select between policy alternatives. We present findings for each of these themes in turn.

**Table 2:** Respondent characteristics

Variable	***n*** (%)	***n*** (%)^**b**^
Mid-level manager demographics	Screening and assessment decisions^**a**^ (***n*** = 32)	Complete sample (***n*** = 90)
**Gender**		
F	24 (75)	72 (80)
M	8 (25)	18 (20)
**Race/ethnicity**		
Non-Hispanic White	23 (72)	65 (72)
Non-Hispanic Black	1 (3)	4 (4)
Unreported	8 (25)	21 (23)
**Agency**		
Child Welfare	19 (59)	46 (51)
Mental Health	4 (13)	19 (21)
Medicaid	3 (9)	11 (12)
Other	6 (19)	14 (16)
**Region**		
North-East	7 (22)	21 (23)
Mid-West	11 (34)	27 (30)
South	6 (19)	13 (14)
West	8 (25)	29 (32)

^a^Screening and assessment decisions: respondents who spoke specifically to decisions regarding the trauma-specific screening, assessment, and treatment.

^b^For the complete sample, percentage totals, in two cases (i.e. race/ethnicity and region) total to 99% due to rounding

### The inter-related decision set and choices considered

Policymakers articulated a set of specific and inter-related decisions required to develop system-wide trauma-focused approaches to screen and assess children entering foster care. Across interviews, analyses revealed the need for decisions in five domains, including (a) reach, (b) content of the endorsed screening tool, (c) the threshold or “cut-score” to be used, (d) the resources to start-up and sustain a protocol, and (e) the capacity of the service delivery system to respond to identified needs. Table 3 provides a summative list of choices articulated across all respondents who reported respective decision points within each of the five domains. All decision points presented more than one choice alternative, with respondents articulating as many as six potential alternatives for a single decision point (e.g., administrator credentials).

**Table 3:** The inter-related decision set for trauma screening, assessment, and treatment

Range of decisions considered	Choices considered	Illustrative quote
**REACH of the screening protocol**		
**Target population**: Whether to screen the entire population or a sub-population with a specific screening tool	Who to screen, specifically whether to implement: 1. Universal population-level screening for all children entering foster care 2. Universal screening for a subsample of children entering foster care 3. No universal screening administered	“We had one county partner in our area that said any kid that comes in, right, any kid who we accept a child welfare referral on we will do a trauma screen on, and then if needed that trauma screen will get sent over to a mental health provider for a trauma assessment and treatment as needed. That was great and that's not something that all of our county partners were able to do just based on capacity, and frankly, around – some of our partners at mental health don't – I don't want to say they don't have trauma-informed providers – but don't have the capacity to treat all of our kids that come in Child Welfare's door with trauma-informed resources.”—Child Welfare
**Timing of initial screening**: When should the screener be initially administered?	The timeframe within which the screening is required, specifically whether the screening must be complete within: 1. 7 days of entry into foster care 2. 30 days of entry into foster care 3. 45 days of entry into foster care	“Not – we had to move our goal of the time when we were going to – so the time limit or – what's the – timeline for having the screeners done used to be 30 days. And we had to move it out to 45 days. So that's more on the pessimistic side, I guess, because I'd rather us be able to do quality work of what we can do, rather than try to overstretch and then it just be shoddy work that doesn't mean anything to anybody.”—Child Welfare
**Ongoing screening**: Determine whether to and the frequency for when to rescreen for trauma	The frequency within which a screening must be re-administered; options included: 1. Screening completed only at entry 2. Screening completed every 30 days 3. Screening completed every 90 days 4. Screening completed every 180 days 5. Screening completed at provider’s discretion when significant life events occur	“Interviewee: Well, we had to make the determination of frequency of the measure that was being implemented and what really seems the most practical and logical. Some of the considerations that we were looking at first and foremost was, like what level of frequency would give us the most beneficial data that we could really act upon to make informed decisions about children's behavioral health needs? And recognizing that this is not static, it's dynamic.”—Other State Partner
**Collaterals**: Who should be a collateral consulted in the screening process (e.g., caregiver of origin, foster parent, etc.)?	The collaterals who might inform the screening process; options include: 1. Caregivers of origin and foster parents 2. Caregivers of origin only 3. Foster parents only	“Another difficulty we’ve had is always including birth families at these assessments. We feel that’s integral, especially for a temporary court ward, to get that parent’s perspective, to help this parent understand the trauma.”—Child Welfare
**Content of the screening tool**		
**Construct**: Whether screen would assess adequately for trauma exposure and/or symptoms as defined by the agency	The content of the screening tool; options include screening of: 1. Both trauma symptomology and exposure 2. Trauma symptomology 3. Broad trauma exposure 4. Specific trauma exposure (e.g., war, sexual abuse)	“But I think that we're interested more in – and when I talk to the Care for Kids, they do not have, beyond that CANS screen, a specific trauma screen. But I think what we're most interested in is not a screening for trauma overall but screenings specifically for trauma symptomatology ‘cause I think almost by definition, foster care children are going to have high trauma scores.”—Medicaid
**Discretion**: Whether to mandate a single tool for screening trauma or provide a list of recommended screening tools	The extent of discretion provided to the clinician in assessing trauma; options include: 1. State mandated screening tool 2. County mandated screening tool 3. List of potential screeners required by state 4. Provider discretion	“Yeah. You know how we have endorsed screening tools is we believe there are psychologists, psychiatrists out there that actually they’re doing the screening and physicians. And as long as it is like a reputable tool, like you said, if they have a preference, and I don’t know the exact numbers but there might be let’s say five tools that are really good for picking up trauma in children. And we say to the practitioner if there's on that you like better over the other we’re giving you the freedom to choose as long as it's a validated tool.”—Mental Health
**Threshold (often known as “cut-score”) of the screening tool**		
**Threshold-level**: Identify whether to adjust the screening threshold (i.e., “cut-score”) and if so, what threshold would be used for further “referral”	The “cut-score” or threshold used for referring to additional services; options included: 1. Set threshold at developer recommendation 2. Set threshold above developer recommendation	“And so we did consider things like that. Maybe the discussion was we really would like to go with a six and above, but you know what, we're gonna go with an eight and above because capacity probably, across the state, is not gonna allow us to do six and above.”—Mental Health
**Mandated “Cut-Score**:**”** Identify whether implement a statewide or county-specific “cut-score”?	Option include: 1. Statewide “cut-score” required 2. Statewide minimal standard set with county-level discretion provided 3. Complete county discretion	“What their threshold is in terms of what is an appropriate screen in referral, that can also vary county to county. And the reason for those business decisions was the service array varies hugely between the counties in Colorado, available service array, and they didn't want to be overwhelming their local community mental health centers with a whole bunch of referrals that all come at once as child welfare gets involved. So the idea was to let the counties be more discriminating on how they targeted this intervention.”—Child Welfare
**Additional criteria for referral**: Identify whether referrals are advanced based on scores alone, concerns alone, or both	Options for materials supplemental to the cut-score, alone, included: 1. Referrals advanced based on scores alone 2. Referrals based on provider/caseworker concern alone 3. Referrals based on either scores or provider/caseworker concern	“Really what we decided, or what we’ve kind of discussed as far as thresholds is not necessarily focusing so much on the number. We do have kind of a guide for folks to look at on when they should refer for further assessment. But we really focused heavily on convening a team on behalf of the child. I know that you’ll talk with [Colleague II] who is our My Team manager. So [state] has a case practice model called My Team. It’s teaming, engagement, assessment, and mentoring. So those are kind of the four main constancies of that model.”—Child Welfare
**Resources to start-up and sustain the screening tool**		
**Administrator credentials**: Who can administer the screening?	Options for required credentials to administer the screening included: 1. Physician 2. Physician with trauma training/ certification 3. Masters-level clinicians 4. Masters-level clinician with trauma training/certification 5. Caseworkers 6. Caseworkers with trauma training/certification	“Yes. So we have, like I said, a draft trauma protocol that we have spent a lot of time putting together. So some of the areas on that are the administration of the checklist. That was kind of based on the instructions that we received from CTAC since they developed the tool. That it’s really staff that administer the tool, they do not need to have a clinical, necessarily, background to be able to administer that tool since it’s just a screening.”—Child Welfare
**Administrator training**: What training is required to implement the screening tool and what are the associated fiscal and human resources required for implementation?	Options for the training resources required included: 1. Training for all child welfare staff on screening 2. Training for new child welfare staff 3. Training for specialized sub-population of child welfare staff	“Several of our counties had already been trained by [CTAC Developer] to use the screening tool, but it wasn’t something that we were requiring. So this will, it’s now going to be a mandatory training for all public and private child welfare staff to administer that screening to all kids in care.”—Child Welfare
**Capacity of service delivery system to respond with a trauma-specific service array**		
**Development of trauma-specific service array**: Are trauma treatments mandated or is a list of recommended treatments provided?	Options for the development of a trauma-specific service array included: 1. Selection of trauma treatments to accommodate available workforce capacity 2. Increase of the threshold for screening treatment to decrease required workforce capacity	“So we don't require any particular instrument, we just require that they address the trauma and that if they are doing – if they say that they are doing trauma therapy or working with a trauma for a kid, that therapist has to be trauma certified. We have put that requirement. So it's kind of hitting it from both ends.”—Other State Partner
**Development of new capacity**: How to build new capacity in the workforce to address anticipated trauma treatment needs?	Options to build the capacity of trauma-specific services included: 1. Single mandated tool required 2. List of new psychosocial services to address trauma 3. Requirement that therapist must be trauma certified	“And so those were some of the drivers around thinking about TARGET as a potential intervention that – because it doesn't require licensed clinicians to provide the intervention because it is focused on educating youth around the impact of exposure to trauma, the concept of triggers, recognizing being triggered and enhancing their skills around managing those responses.”—Child Welfare

### Information continuum to inform understandings of options’ success and failure

**Fig. 2:**
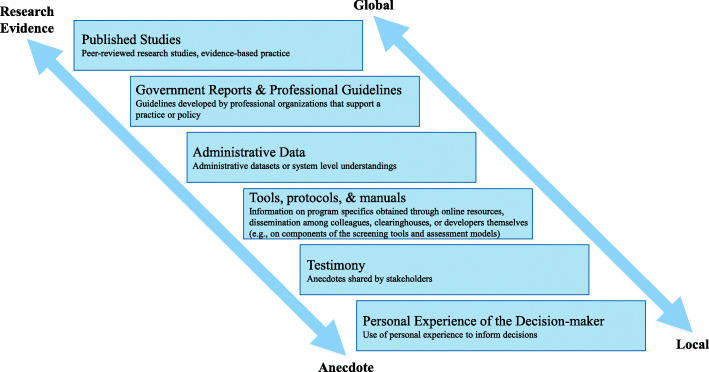
Information from Research Evidence to Anecdote

To inform decisions, mid-level managers sought information from a variety of different sources ranging from research evidence (e.g., descriptive studies of trauma exposure, intervention or evaluation studies of practice-based and system-wide screening initiatives, meta-analyses of available screening and treatment options, and cost-effectiveness studies [3]) to anecdotes (see Fig. 2). Across this range, information derived from both global contexts, defined as evidence drawn from populations and settings outside of the jurisdiction, and local contexts, defined as evidence drawn from the affected populations and settings [50]. Notably, systematic research evidence most often derived from global contexts, whereas ad hoc evidence most often derived from local sources.

### Evidence gaps

Respondents reported that the amount and type of information available depended on the decision itself. Comparing the decision set with the types of information available facilitated identification of evidence gaps. For example, respondents reported far more research literature about where a tool’s threshold should be set (based on its psychometric properties) than about the resources required to initiate and sustain the protocol over time. As a result, ad hoc information was often used to address these gaps in research evidence. For example, respondents indicated greater reliance on information such as testimony and personal experience to choose between policy alternatives. In particular, respondents often noted gaps in generalizability of research evidence to their jurisdiction and relevant stakeholders. As one respondent at a mental health agency notes, “I found screening tools that were validated on 50 people in [another city, state]. Well, that's not the population we deal with here in [our state] in community mental health or not 50 kids in [another city]. I want a tool to reflect the population we have today.”

**Fig. 3:**
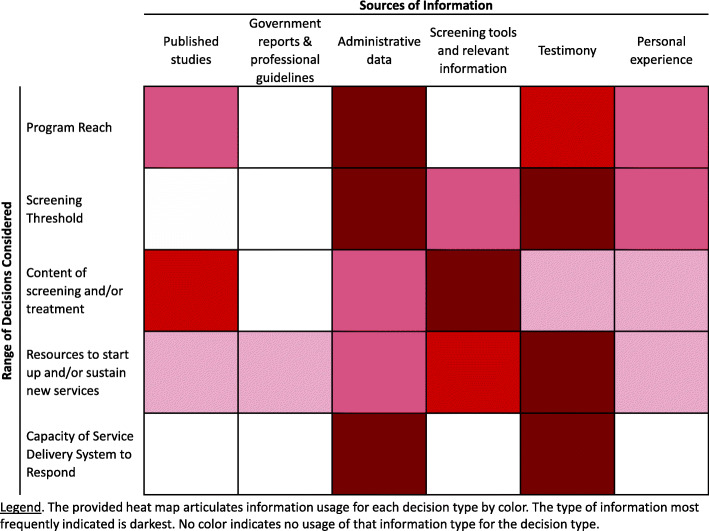
Information Gaps across the Dynamic Decision Continuum

The decision sampling method revealed where evidence gaps were greatest, allowing for further analysis to identify whether these gaps were due to a lack of extant evidence or the accessibility of existing evidence. In Fig. 3, a heat map illustrates where evidence gaps were most frequently expressed. Notably, this heat map illustrates that published studies were available for each of the relevant domains except for the “capacity for delivery systems to respond.”

### Role of expertise in assessing and contextualizing available research evidence

As articulated in Table 4, respondents facing decisions not only engaged with information directly, but also with professionals holding various kinds of clinical, policy, and/or public sector systems expertise. This served three purposes. First, respondents relied on experts to address information gaps. As one respondent illustrates, “We really found no literature on [feasibility to sustain the proposed screening protocol] … So, that was where a reliance in conversation with our child welfare partners was really the most critical place for those conversations.” Second, respondents relied on experts to help sift through large amounts of evidence and present alternatives. As one respondent in a child welfare agency indicates:

“I would say the committee was much more forthright… because of their knowledge of [a specific screening] tool. I helped to present some other ones … because I always want informed decision-making here, because they've looked at other things, you know...there were people on the committee that were familiar with [a specific screening tool]. And, you know, that's fine, as long as you're – you know, having choice is always good, but [the meeting convener] knew I'd bring the other ones to the committee.”

**Table 4:** Types of expertise across the decision set

Expertise	Operational definition	Types of policy decisions expertise is used to inform	Illustrative quote
Clinical expertise	Knowledge gained from collaborations with individuals who have expertise in medicine (e.g., physicians, psychiatrics, psychologists, community mental health providers, developers who have a clinical background)	• Reach • Threshold • Content of screening • Resources to start-up and/or sustain screening, assessment, and/or treatment protocol	“So for rescreening, when we were discussing it we have kind of a steering committee that we’ve used. A variety of folks, some… partners and some folks from our field that we used to kind of pull together to help make some of these decisions.”—Child Welfare
Policy expertise	Knowledge gained from collaborations with individuals who have expertise in policy development	• Content of screening • Resources to start-up and/or sustain screening, assessment, and/or treatment protocol	“Yeah, I think so. I think because there were a few people at the table who had and have policy backgrounds. So, there was certainly – one of the members of the team, I know, had been actively involved in an analyst involved in writing foster care policy.”—Child Welfare
System expertise	Knowledge gained from collaborations with individuals who have expertise in public sector systems including the child welfare, Medicaid, or mental health systems (e.g., caseworkers, other state child welfare offices, child welfare partners, mental health workforce)	• Reach • Threshold • Content of screening • Resources to start-up and/or sustain screening, assessment, and/or treatment protocol • Capacity of service delivery system to respond	“We really found no literature on that when it came to – I mean; I think because this is such a unique thing that we're doing in comparison to other states that we were really blazing our own path on that one. So, that was where a reliance in conversation with our child welfare partners was really the most critical place for those conversations.”—Other State Partners

Third, respondents reported reliance on experts to assess whether research evidence “fit to context” of the impacted sector or population. As one mental health professional indicated:

“It just seems to me when you're writing a policy that's going to impact a certain areas’ work, it's a really good idea to get [child welfare supervisor and caseworker] feedback and to see what roadblocks they are experiencing to see if there's anything from a policy standpoint that we can help them with…And then we said here's a policy that we're thinking of going forward with and typically they may say yes, that's correct or they'll say you know this isn't going work have you thought about this? And you kind of work with them to tweak it so that it becomes something that works well for everybody.”

Therefore, clinical, policy, or public sector systems expertise played at least three different roles—as a source of information in itself, as a broker that distilled or synthesized available research evidence, and also as a means to help apply (or assess the “fit” of) available research evidence to the local context.

### The role of values in selecting policy alternatives

When considering choices, respondents focused on their associated benefits, challenges, and “trade-offs.” Ultimately, decision-makers revealed how values drove them to select one alternative over another, including commitments to (a) a trauma-informed approach, (b) aligning practice with the available evidence base, (c) aligning choices with available expert perspectives, and (d) determining the capacity of the system to implement and maintain.

For example, one common decision faced was where to set the “cut-off” score for trauma screening tools. Published research typically provides a cut-off score designed to optimize sensitivity (i.e., the likelihood of a true positive) and specificity (i.e., likelihood of a true negative). One Medicaid respondent engaged a researcher who developed a screening tool who articulated that the evidence-based threshold for further assessment on the tool was a score of “3.” However, the public sector decision-maker chose the higher threshold of “7” because they anticipated the system lacked capacity to implement and respond to identified needs if set at the lower score. This decision exemplifies a trade-off between being responsive to the available evidence base and system capacity. As the Medicaid respondent articulated: “What we looked at is we said where we would we need to draw the line, literally draw the line, to be able to afford based on the available dollars we had within the waiver.” The respondent confronted a trade-off between maximizing effectiveness of the screening tool, the resources to start-up and sustain the protocol, and the capacity of the service delivery system to respond. The respondent displayed a clear understanding of these trade-offs in the following statement: “…children who have 3 or more identified areas of trauma screen are really showing clinical significance for PTSD, these are kids you should be assessing. We looked at how many children that was [in our administrative data], and we said we can’t afford that.” In this statement, consideration for effectiveness is weighed against feasibility of implementation, including resources to initiate and sustain the policy.

## Discussion

By utilizing a qualitative method grounded in the decision sciences, the decision sampling framework provides a new methodological approach to improve the study and use of research evidence in real-world settings. As the case study demonstrates, the approach draws attention to how decision-makers confront sets of inter-related decisions when implementing system-wide interventions. Each decision presents multiple choices, and the extent and types of information available are dependent upon the specific decision point. Our study finds many distinct decision points were routinely reported in the process of adoption, implementation, and sustainment of a screening program, yet research evidence was only available for some of these decisions [51], and local evidence, expertise, and values were often applied in the decision-making process to address this gap. For example, decision-makers consulted experts to address evidence gaps and they relied on both local information and expert judgment to assess “fit to context”—not unlike how researchers address external validity [52].

Concretely, the decision sampling framework seeks to produce insights that will advance the development of strategies to expedite research evidence use in system-wide innovations. Similar to strategies used to identify implementation strategies for clinical intervention (e.g., implementation mapping) [53], the decision sampling framework aims to provide evidence that can be informative to identification of the strategies required to expedite adoption of research findings into system-wide policies and programs. Notably, decision sampling may also identify evidence gaps leading to the articulation of areas where new lines of research may be required. Whether informing implementation strategies or new areas for research production, decision sampling roots itself in understanding the experience and perspectives of decision-makers themselves, including the decision set confronted, the role of various types of information, expertise, and values influencing the relevant decisions. Analytic attention on the decision-making process leads to at least three concrete benefits as articulated below.

First, the decision sampling framework challenges whether we refer to a system-wide innovation as “evidence-based” rather than as “evidence-informed.” As illustrated in our case example, a system-wide innovation may engage some decisions that are “evidence-based” (e.g., selection of the screening tool) while other decisions fail to meet the criteria whether because of lacking access to a relevant evidence base, expertise, or potentially holding values that prioritized other considerations over the research evidence alone. For example, respondents in some cases adopted an evidence-based screening tool but then chose not to implement the recommended threshold; while thresholds are widely recommended in peer-reviewed publications, neither their psychometric properties nor the trade-offs they imply are typically reported in full. Thus, the degree to which screening tools are based on “evidence derived from applying systematic methods and analyses” is an open question [54, 55]. In this case, an element of scientific uncertainty may have driven the perceived need for adaptation of an “evidence-based” screening threshold.

Second, decision sampling contributes to theory on why studies of evidence use should address other sources of information beyond systematic research [56]. Our findings emphasize the importance of capturing the heterogeneity of information used by decision-makers to assess “fit to context” and address the scientific uncertainty that may characterize any one type of evidence alone [14]. Notably, knowledge synthesis across the information continuum (i.e., research evidence to ad hoc information) may facilitate the use of available research evidence, impede it, or complement it, specifically in areas where uncertainty or gaps in the available research evidence exist. Whether use of other types of information serves any of these particular purposes is an empirical question that requires further investigation in future studies.

Third, the decision sampling framework makes a methodological contribution by providing a new template to produce generalizable knowledge about quality gaps that impede research evidence use in system-wide innovations [17] as called for by Gitomer and Crouse [57]. By applying standards of qualitative research for sampling, measures development, and analysis to mitigate potential bias, this framework facilitates the production of *knowledge* that is *generalizable* beyond any one individual system, as prioritized in implementation sciences [1]. To begin, the *decision sampling* framework maps the qualitative semi-structured interview guide on tenets of the decision sciences (see Fig. 1). Integral to this approach is a concrete cross-walk between the theoretic tenets of decision sciences and the method of inquiry. Modifications are possible; for example, we engaged individual interviews for this study but other studies may warrant group interviews or focus groups to characterize collective decision-making processes. Finally, our measures align with key indicators in decision analysis, while other studies may benefit from crosswalks with other theories (e.g., cumulative prospect theory to systematically examine heuristics and cognitive biases) [58]. Specific cases may require particular attention to one or all of these domains. For example, an area where the information continuum is already well-documented may warrant greater attention to how expertise and values are drawn upon to influence the solution selected.

Fourth, decision sampling can help to promote the use of research evidence in policy and programmatic innovation. Our findings corroborate a growing literature in the implementation sciences that articulates the value of “knowledge experts” and opinion leaders to facilitate innovation diffusion and research evidence use [56, 59]. Notably, such efforts may require working with decision-makers to understand trade-offs and the role of values in prioritizing choices. Thus, dissemination efforts must move beyond simply providing “clearinghouses” that “grade” and disseminate the available research evidence and instead engage knowledge experts in assessing fit-to-context, as well as the applicability to specific decisions. To move beyond knowledge transfer, such efforts call for novel implementation strategies, such as group model building and simulation modeling, to assist in consideration of the trade-offs that various policy alternatives present [60]. Rather than attempting to flatten and equalize all contexts and information sources, this method recognizes that subjective assessments, values, and complex contextual concerns are always present in the translation of research evidence to implementation.

Notably, evidence gaps also demand greater attention from the research community itself. As traditionally conceptualized, research evidence arrives from academic centers that design and test evidence-based practices prior to dissemination; this linear process has been cited as the “science push” [60] to practice settings. In contrast, our findings support models of “linkage and exchange,” emphasizing the bi-directional process by which policymakers, service providers, researchers, and other stakeholders work together to create new knowledge and implement promising findings [60]. Such efforts are increasingly prioritized by organizations such as the Patient-Centered Research Outcomes Institute and the National Institutes of Health which have called for every stage of research to be conducted with a diverse array of relevant stakeholders, including patients and the public, providers, purchasers, payers, policymakers, product makers, and principal investigators.

### Limitations

Any methodological approach provides a limited perspective on the phenomenon of interest. As Moss and Hartel (2016) note “both the methods we know and the methodologies that ground our understandings of disciplined inquiry shape the ways we frame problems, seek explanations, imagine solutions, and even perceive the world.” (p. 127) We draw on the decision sciences to propose a decision sampling framework grounded in the concept of rational choice as an organizing framework. At the same time, we have no expectation that the machinations of the decision-making process align with the components required of a formal decision analysis. Rather, we think engaging a decision sampling framework facilitates characterizing and analyzing key dimensions of the inherent complexity of the decision-making process. Systematic collection and analysis of these dimensions is proposed to more accurately reflect the experiences of decision-makers and promote responsive translational efforts.

Characterizing the values underlying decision-making processes is a complex and multi-dimensional undertaking that is frequently omitted in studies on research evidence use [15]. Our methodological approach seeks to assess values both by asking explicit questions and by evaluating which options “won the day” as respondents indicated why specific policy alternatives were selected. Notably, values as articulated and values as realized in the decision-making process frequently did not align, offering insights into how we ascertain information about the values of complex decision-making processes.

## Conclusions

Despite increased interest in research evidence use in system-wide innovations, little attention has been given to anchoring studies in the decisions, themselves. In this article, we offer a new methodological framework for “decision sampling” to facilitate systematic inquiry into the decision-making process. Although frameworks for evidence-informed decision-making originated in clinical practice, our findings suggest utility in adapting this model when evaluating decisions that are critical to the development and implementation of system-wide innovations. Implementation scientists, researchers, and intermediaries may benefit from an analysis of the decision-making process, itself, to identify and be responsive to evidence gaps in research production, implementation, and dissemination. As such, the decision sampling framework can offer a valuable approach to guide future translational efforts that aim to improve research evidence use in adoption and implementation of system-wide policy and programmatic innovation.

## Supplementary Information

**Additional file 1.**

## Data Availability

Due to the detailed nature of the data and to ensure we maintain the confidentiality promised to study participants, the data generated and analyzed during the current study are not publicly available.
