# Supplementary material for: The decision sampling framework: a methodological approach to investigate evidence use in policy and programmatic innovation
Source: Implement Sci. 2021 Mar 11;16:24. doi: 10.1186/s13012-021-01084-5 (PMC7953669; doi:10.1186/s13012-021-01084-5)
Supplement: Supplementary file 1 — Additional file 1. [file 13012_2021_1084_MOESM1_ESM.docx]

**The Decision Sampling Framework: A Methodological Approach to Investigate Evidence Use in Policy and Programmatic Innovation**

**Supplementary Materials**

| **Content** | | **Page number** |
| --- | --- | --- |
| S-1 | Semi-structured Interview Guide: Selected Excerpts for “Decision Sampling” | 2-3 |
| S-2 | Decision Sampling Data Matrix | 3-4 |
| S-3 | Scientific Rigor | 4 |

**S-1. Semi-Structured Interview Guide: Excerpts for Decision Sampling**

**B3. Anchoring and confirming specific case.**

B3a. Within this involvement, what services to identify or treat trauma were you most recently involved with *prioritizing, selecting, implementing or evaluating* for your state?

B3b. We would like to focus the following question on this service in particular. Please let us know if there is another example that you would prefer to speak about.

Yes. *If so,* which services would you prefer to speak about? What was your role in relation to [*prioritization of the concern*, *selection/implementation/sustaining]* of this service in particular? *Probe:* Do you have any documents that describe the approach that you are taking and/or rationale for it? *If so,* would you be able to share this with us? (*Open-ended)*

No. What was your role in relation to prioritization*/selection/implementation/sustaining]* of this service in particular?

B3c. As you think about the process for [*prioritizing/selecting/implementing/sustaining*] the [*trauma-informed service specified in B3*], what decisions do you recall the group needing to make? [*Interviewer summarizes each decision identified.*] *Probe:* Of these, which did you think was the most important decision? How come? What was the most recent important decision that you can comfortably recall made by the group in which you were involved?

**B4. Context for decision making.**

B4a. Briefly, please describe the process the group took to make [the most recent important decision you were involved in, referred to in B3c.]? [*Probe:* What was the focus for convening the group? How were decisions made? Who led this process? As leader within your system, what is your approach to managing this type of process? How do you use information to assist in this process? *Interviewer probes on the focus and power/authority of meetings.*

B4b. Who participated in the group process?

B4c. How did communication occur for this decision? [*Probe:* for [type of communication], did this occur routinely or ad hoc? With what frequency did this occur?] [*Interviewer focuses on formality, frequency, and format of correspondence, checking all that apply*]

B4d. Who was involved in making [the ultimate decision reached, referenced in B3c] specifically? [*Probe:* Who was involved in this process?]

B4d. What was your role in [this decision]? Probe: Were you able to inform [the decision] made? If so, how?

**B5. Decision criteria and thresholds individually.**

B5a. For you personally, what were the most important factors in your decision-making process?

B5ai. Every person has certain values that guide our decisions. Sometimes these may be unspoken and in other cases communicated to others participating in the process. How did your own values influence this process? *Probe:* What outcomes in [*prioritizing/selecting/implementing/sustaining*] the [*trauma- informed services]* were of greatest importance to your own decision making process?

B5b. When you were personally seeking to identify how best to proceed on [*specify most recent decision*], did you consider more than one solution? If so, which ones?

B5bi. For you personally, what did you see as the benefits of the potential solution(s)? For you personally, what did you see as the drawbacks for the potential solution(s)?

B5bii. How did you weigh the benefits and drawbacks of the solution(s)? How did this inform your ultimate decision as how to best proceed?

**B6. Decision-making process.**

B6a. In making this decision, where did you go to find out information to inform the decision?

B6b. Based on the information you listed, what type of information was *most* useful? How come? Where did this information come from? Why did you select this evidence, in particular? What aspects of the information led you to prioritize its use over types of evidence? What type of information was *least* useful? How come?

B6c. When you think about all the different types of information you gathered during the decision-making process, how did you put it all together and make sense of it?

B6d. Who was involved in helping to make sense of all the information?

B6di. Do you think you had enough information to make this decision?

Yes. *If yes,* when did you know you had enough information?

No. *If no,* what information would you have wanted that you did not receive?

B6dii. Do you think this was an evidence-informed decision?

Yes.

No. *If no,* why not?

Do not know.

B6diii. Did the provided information present any uncertainty as to what the optimal approach would be?

B6e. What expertise, if any, did you seek out to help you understand, interpret, or evaluate the information that was available?

B6f. In reflecting on this decision, what ultimately determined the group’s decision?

**S-2. Decision Sampling Data Matrix**

| **Code #** | **Code** | **Interviewer’s Inductive Thematic Summary** | **Illustrative Quotes** |
| --- | --- | --- | --- |
| **N/A** | Participant ID and Information | Participant ID:  Decision Type:  Decision Stage:  Decision: Categorization: |  |
| **N/A** | Demographic Survey Data | Job Title:  Office:  Job History within agency:  Educational background: |  |
| **104.0** | Respondent role in trauma-informed services [Role of respondent in trauma-informed services] |  |  |
| **1.0** | Decision Point 1 |  |  |
| **1.1** | Catalyst for Decision |  |  |
| 1.2 | Stage of Policy/Programmatic Development for Decision Point 1 |  |  |
| **1.3** | Options considered & not selected, with rationale |  |  |
| **1.4** | Any adaptation to established approach for customization to state systems |  |  |
| **1.5** | Type, extent, and frequency of meeting for group involved in decision-making process |  |  |
| **1.5a** | Governance structure of decision [provided input, made decision, notified of decision] |  |  |
| **1.6** | Respondent role in decision |  |  |
| **1.7** | Factors influential to Decision-making Process, including sociopolitical context |  |  |
| **1.8** | Types of Information and sources of information |  |  |
| **1.8a** | Most useful information |  |  |
| **1.8b** | Least useful information |  |  |
| **1.8c** | Sensemaking of information |  |  |
| **1.9** | Role of values in Decision-making process |  |  |
| **1.10** | Perceived adequacy of available information to make decision |  |  |
| **1.11** | Determination of whether or not an evidence informed decision |  |  |
| **1.12** | Limitation in evidence base, itself |  |  |
| **1.13** | Resolution reached |  |  |
| **1.14** | Decision Threshold |  |  |
| **1.15** | How evidence is used |  |  |

**S-3. Member-checking Focus Group Guide**

[Study findings were initially presented in a slide presentation prior to questions being asked below]

1. Does this match your experience?
2. Do you want to change anything?
3. Do you want to add anything?

[A Monte Carlo simulation model was presented and then the following questions were asked]

1. We developed a system model to help policymakers integrate state/county context to address questions regarding reach, screening content, screening threshold, and system capacity. Could this type of model be helpful to your work?
2. To apply your work, what would need to be different about the model? Probe: Parameters? Structures?
3. Do you have any administrative data that could help to inform this process?
4. Would this model make you rethink your assumptions? Do you think everyone in your system has the same perspectives of tradeoffs?
5. If an expert team were available to work with you and others from your system, do you think that a modeling effort like this could help you plan your screening policies?
